# Anisotropy in Creep Behavior of a Directionally Solidified Ni-Based Superalloy at 980 °C and 1070 °C

**DOI:** 10.3390/ma18091998

**Published:** 2025-04-28

**Authors:** Anping Long, Xiaoshan Liu, Junyi Cheng, Jiangying Xiong, Ganjiang Feng, Jianzheng Guo, Rutie Liu

**Affiliations:** 1 State Key Laboratory of Powder Metallurgy, Central South University, Changsha 410083, China; 193302073@csu.edu.cn (A.L.); liuxs@wedge.com.cn (X.L.); 223302084@csu.edu.cn (J.C.); 203302086@csu.edu.cn (J.X.); fenggj@wedge.com.cn (G.F.); 218062@csu.edu.cn (J.G.); 2 Wedge Central South Research Institute Co., Ltd., Shenzhen 518045, China

**Keywords:** superalloys, directionally solidified, anisotropy, creep mechanisms, microstructure evolution

## Abstract

Directionally solidified (DS) superalloys have become a primary material choice for turbine blade applications. Due to the complex shape of the blades, certain regions inevitably experience stress axes oriented orthogonally to the crystal growth direction. Therefore, this study explores the creep characteristics of a DS superalloy in different orientations (transverse (T) versus longitudinal (L) with respect to grain growth direction) under intermediate and high temperatures (980 °C and 1070 °C), while simultaneously analyzing their respective deformation mechanisms and microstructural transformation behaviors. Experimental findings reveal pronounced orientation-dependent variations in creep performance, deformation modes, and microstructural development. Notably, the T specimen exhibits higher creep resistance at 980 °C, which can provide a basis for the design of some components that require high creep resistance and maintain small deformation. At 980 °C, L specimens primarily undergo γ′ phase shearing via antiphase boundaries (APBs) pairs, whereas T specimen exhibits APB pairs and superlattice intrinsic stacking faults (SISFs) shearing mechanisms. At 1070 °C, the L specimen exhibits dislocation shearing of γ′ alongside dislocation bypassing of tertiary γ′, while the T specimen demonstrates dislocation climbing within the γ channels. Additionally, the L specimen exhibits significant N-type rafting, while the T specimen shows significant Ostwald ripening characteristics, with an Ostwald ripening rate constant of 1.04 × 10^−20^ m^3^/h.

## 1. Introduction

Modern aero-engines demand elevated inlet temperatures to achieve superior thrust-to-weight ratios and fuel economy, yet this imposes stringent requirements on material performance under high-temperature conditions [1,2,3,4,5,6]. To address this, single-crystal or DS superalloys are widely employed, as their absence of grain boundaries along the primary stress axis significantly enhances resistance to high-temperature creep. Single-crystal superalloys have disadvantages such as high cost, susceptibility to spurious crystal freckles, and low yield of complex shaped parts [7,8,9], making DS superalloys a compromise. Ni-based DS superalloys like CM247LC, PWA1426, and IC10 are prevalent in aero-engine turbine blade manufacturing [10,11,12].

DS superalloys consist of rod-shaped grains oriented in the growth direction [001], while the orientation perpendicular to the growth direction is random. The creep mechanism of DS superalloys along the longitudinal direction is similar to that of single crystals and has been extensively studied [13,14,15,16,17,18,19]. These studies have reported that dislocations bypass, cross-slip, climb, or shear through the γ′ precipitates during their motion. Among these, the shearing of γ′ occurs in various forms under different temperature and stress conditions. For example, Knowles D. and Li, J.C. [14,16] found that in high-stress creep regimes, the deformation usually proceeds via stacking fault shearing. Eggeler G. [18] discovered that in CMSX-6 alloy in low-stress creep regimes (1025 °C/80 MPa), multiple <110>{111} slip systems are activated, and the γ′ precipitates are sheared by dislocations, creating anti-phase boundaries (APBs). Guo J.T. [15] found in the creep study of DZ17G alloy that the creep mechanism in low-stress regimes is dislocation climb. These findings indicate that the creep deformation mechanisms are diverse under different conditions and need to be analyzed specifically according to the actual situation.

Despite abundance of research regarding creep properties of DS superalloys, there are still few studies on the creep anisotropy [20,21,22,23]. The existing studies mostly focused on how creep life differs depending on orientation and the effects of various contributing elements. For example, Stinville, J.C. and Mataveli S.L. [20,21] investigated the creep behavior of a DS superalloy in different orientations and found that the transverse creep life was lower than the longitudinal at all temperatures. Research indicated that crystallographic structure, high-angle grain boundaries, and grain boundary oxidation were the main factors contributing to the lower transverse creep life. Similar patterns were reported in this study. However, this paper further explores the intrinsic mechanisms of the differences in creep properties from the perspective of dislocation motion caused by crystallographic structures. It is significant for fully understanding the creep deformation behavior and corresponding micro-mechanisms under different orientations. This paper compares minimum creep rates and stress exponents and finds that although creep life is lower in transverse than that of longitudinal, the transverse minimum creep rate is lower than the longitudinal direction under all stress conditions at 980 °C. It indicates that the transverse orientation has higher creep resistance, but the lower elongation capability leads to a lower creep life. This observation proves highly valuable for designing high-temperature components that focus on high creep resistance. This paper also provides an important reference for understanding the deformation mechanisms in transverse and longitudinal creep of DS nickel-based superalloys, which aids in the orientation design for high-temperature components and improving their service life.

## 2. Materials and Methods

**Figure 1:**
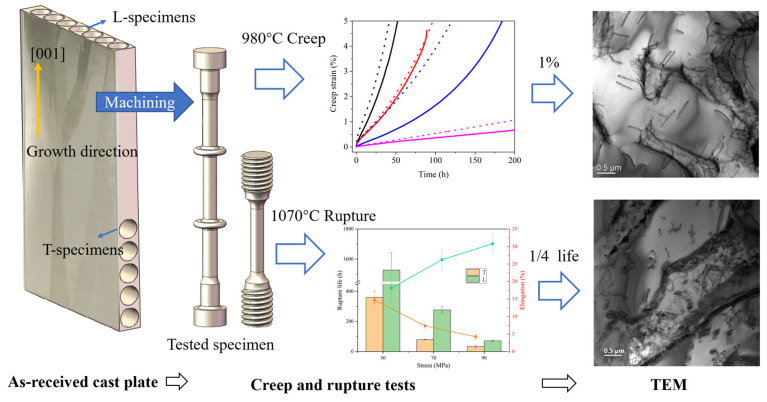
Sampling and testing process diagram. The solid line is for T specimens, dash line for L specimens and color represents stress level in the 980 °C creep curves. The orange represents the T specimens, and green represents the L specimens in 1070 °C rupture comparison.

The research employed an IC10 DS alloy with the following composition (mass fraction, wt.%): Cr 6.5–7.5, Co 11.5–12.5, W 4.7–5.2, Al 5.6–6.2, Ta 6.5–7.5, Mo 1.0–2.0, Hf 1.0–2.0, C 0.07–0.12, B 0.01–0.02, with the balance being Ni. The alloy casting plates were prepared by directional solidification, with dimensions of 120 × 110 × 15 (±0.5) mm. The casting plates were subjected to sup-solvus and aging heat treatment using a Titan (H2) vacuum furnace (Ipsen, Souderton, PA, USA). The thermal treatment began with heating at a rate of 10 °C per minute until reaching 1180 ± 10 °C, where it was maintained for 2 h. Subsequently, the temperature was increased at 5 °C per minute to 1270 ± 10 °C and held steady for another 2 h. The material was then cooled in an argon atmosphere at an approximate rate of 60 °C per minute. For the aging phase, the sample was kept at 1050 ± 10 °C for 4 h before undergoing argon-assisted cooling at around 40 °C per minute. At the sup-solvus temperature, large-sized primary γ′ formed during casting can be dissolved as much as possible; fine and uniformly distributed secondary γ′ can be formed during the subsequent rapid cooling process. The L samples were extracted parallel to the [001] crystallographic orientation of the cast plate, whereas the T specimens were cut orthogonally to this growth axis. Creep testing was carried out at 980 °C with applied stresses varying between 110 MPa and 180 MPa, while rupture tests were executed at 1070 °C under stresses ranging from 50 MPa to 90 MPa. For every testing scenario, three specimens were evaluated: two were tested until failure to generate full creep curves, and the third was interrupted during testing for subsequent TEM analysis. Figure 1 depicts the sampling and experimental procedure. The creep samples featured a flange design to ensure secure extensometer attachment, and their time-dependent strain was monitored using a Heidenhain (Ulm, Germany) extensometer. Both creep and rupture tests employed an RJ-50 creep tester (Kexin, Changchun, China), with temperature control achieved via a resistance-heated furnace.

In order to elucidate the deformation mechanisms exhibited by specimens of different orientations throughout creep and rupture, samples were taken from specimens with a creep elongation of 1% or 1/4 rupture life, and specimens after fracture for microstructural observation. The microstructural features were examined employing a Nikon SMZ1270 optical microscope (OM) (Tokyo, Japan), a Carl Zeiss Sigma300 scanning electron microscope (SEM) (Cambridge, UK), and an FEI Tecnai F20 transmission electron microscope (TEM) (Hillsboro, OR, USA). For OM, SEM, and TEM observations, the samples were extracted from the central region of the specimens’ gauge section. For OM and SEM samples preparation, the surfaces were etched with a solution of 100 mL methanol, 100 mL HCl, and 5 g CuCl_2_. Microstructural analysis was performed via SEM at an accelerating voltage of 20 kV, employing a 120 μm objective aperture. TEM samples were fabricated using an electrical discharge machine (Zhaoming, Shanghai, China) and mechanically grounded to 50 µm thickness, then subsequently thinned via twin-jet polishing in a mixture of 90% CH_3_CH_2_OH and 10% HClO_4_ at 25 °C. Dislocation structures were examined by TEM under varying zone axes and g vectors, with a voltage of 200 kV.

## 3. Results

### 3.1. Original Microstructure

**Figure 2:**
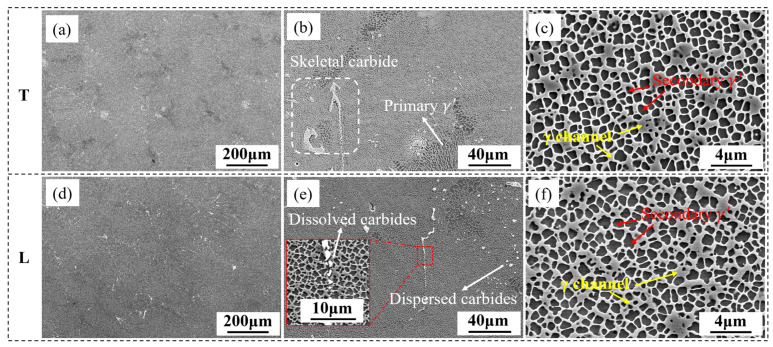
The microstructure of T (**a**–**c**) and L specimens (**d**–**f**) after heat treatment: dendritic stem (**a**,**d**); interdendritic (**b**,**e**); $\gamma^{\prime }$ in dendritic stem (**c**,**f**).

Figure 2 displays the microstructure of the heat-treated alloy casting plate, as previously analyzed in earlier research [24]. The dendritic morphology exhibits minimal variation from T and L samples. Within the dendritic zones, the secondary γ′ phase demonstrates a consistent size distribution, averaging around 810 nm, and exhibits a moderately squared shape. In the course of solution treatment, the large sized γ′ formed in the slow cooling process of casting is dissolved into the matrix. Subsequently, fine and uniformly distributed secondary γ′ precipitates are formed during rapid cooling and further homogenized and squared during the aging process. This fine and uniformly distributed γ′ microstructure with a certain degree of squareness plays a positive role in strengthening the alloy’s resistance to creep deformation [25,26]. A minor quantity of eutectic remains in the interdendritic zones, accompanied by coarse γ′ phase, suggesting incomplete dissolution of the primary γ′ during solution treatment. Besides the eutectic, finely dispersed carbides and coarser skeletal carbides are also observed in these regions. The skeletal carbides show signs of partial dissolution, which lead to the formation of fragmented short rod-shaped carbides, as illustrated in Figure 2e.

### 3.2. Creep/Rupture Properties at 980 °C/1070 °C

**Figure 3:**
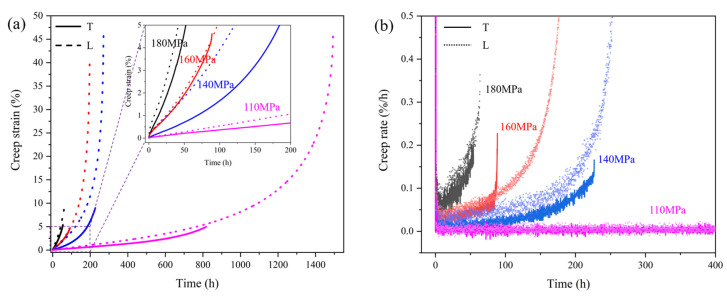
The creep behavior of T and L specimens at 980 °C is illustrated through both strain–time curves (**a**) and corresponding strain rate plots (**b**) across various stress levels, in which the solid line is for T specimens, dash line for L specimens, and color represents stress level.

Figure 3 displays the creep behavior and corresponding strain rate patterns of specimens tested under varying stress levels at 980 °C. The experimental results demonstrate a marked reduction in creep lifespan for both T and L samples with elevated stress, revealing the alloy’s pronounced stress-dependent creep performance at this temperature. By comparing the creep properties of the T and L specimens, the results show that the T specimens have significantly lower creep life and elongation at fracture than the L specimens. Table 1 shows the minimum creep rates obtained in the second stage of creep. The data reveal that the T specimens have lower creep rates than those of the L specimens under all stress conditions, indicating that the T specimens exhibit higher creep resistance under intermediate temperature. This finding can provide a theoretical basis for the design of some hot section components that require high creep resistance and have a low deformation. The deformation mechanism underlying the lower creep rate will be investigated in detail later.

The minimum creep rate depends on stress, temperature, and creep activation energy. The power-law creep equation has been widely used to predict the minimum creep rate of high-temperature alloys [27] and can be expressed as follows:

(1) $$\dot{\varepsilon_{m}}=A{\left(\sigma -\sigma_{p}\right)}^{n}exp(-Q/kT)$$

where $\dot{\varepsilon_{m}}$ stands for minimum creep rate; σ represents the applied stress; A is a constant; $\sigma_{p}$ indicates the frictional stress, which is related to the alloy’s initial γ/γ′ structural configuration; n stands for the stress exponent; Q represents the creep activation energy; k is the Boltzmann constant; and T stands for temperature.

In this study, creep tests at 980 °C were conducted within a relatively low stress range, and the activation energy associated with creep may be considered as approximately equal under different stress conditions. Therefore, the stress exponent n for the T and L specimens at 980 °C can be calculated using the above formula, yielding values of 4.3 and 5.2, respectively. A higher stress exponent indicates a greater sensitivity of the minimum creep rate to stress. According to previous research on stress exponents and creep mechanisms [28,29,30,31], when n ≈ 1, diffusion plays a primary role in the creep process, and when n ≈ 3 to 5, dislocation motion serves as the dominant mechanism governing creep behavior. This suggests that the creep of both T and L specimens at 980 °C is dominated by dislocation motion. Therefore, studying the dislocation motion throughout creep can contributes to an improved understanding of the underlying mechanisms.

**Figure 4:**
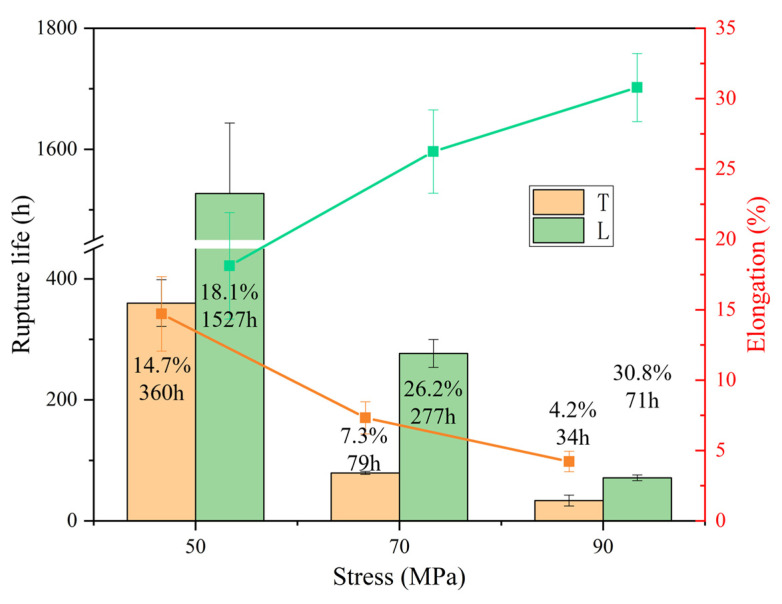
Rupture life (bars) and elongation (lines) of T and L specimens at 1070 °C.

The rupture life and elongation at fracture of T and L specimens at 1070 °C are compared in Figure 4. Under all stress conditions, the rupture life observed in the L specimens is considerably greater than that of the T specimens. At 50 MPa, the rupture life of the L specimen is 4.2 times that of the T specimen, and this ratio decreases to 2.1 times at 90 MPa. The gap between the two gradually decreases with increasing stress, indicating that the L specimen is more sensitive to stress, similar to the situation at 980 °C. The elongation at fracture is also higher for the L specimens than that of the T specimens. As the stress increases from 50 MPa to 90 MPa, the fracture elongation exhibited by the L specimens increases from 18.1% to 30.8%, while that of the T specimens decreases from 14.7% to 4.2%.

### 3.3. Microstructural Characteristics of Fractures

**Figure 5:**
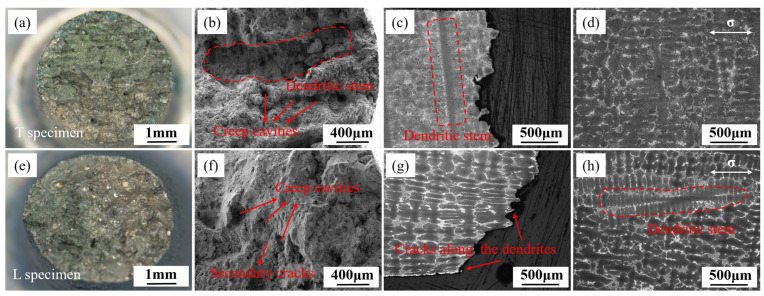
Fracture surface morphology (**a**,**b**,**e**,**f**) and microstructure (**c**,**d**,**g**,**h**) near the fracture of T specimens (**a**–**d**) and L specimens (**e**–**h**) after creep at 980 °C/140 MPa.

Figure 5 illustrate the fracture surface morphology and microstructure near the fracture of T and L specimens under the condition of 980 °C/140 MPa. The fracture surface of the T specimen remains circular, with river-like grooves observable on the fracture surface. The dendritic stem spans the entire fracture surface, and creep cavities are distributed throughout the fracture plane, indicating obvious interdendritic fracture. The fracture surface of the T specimen is parallel to the grain growth direction, and the dendritic stem parallel to the fracture surface can be observed in Figure 5c. Directional solidification forms columnar grains parallel to the heat flow direction (usually the <001> orientation) with dendritic stems. The dendritic stems are typically enriched with elements that enhance solid-solution strengthening (e.g., W, Re, Mo, etc.) and elements that contribute to the formation of γ′ phase (e.g., Al, Ti). The segregation of these elements endows the dendritic stems with a higher solid-solution strengthening effect and hinders dislocation slip through the precipitation of nanoscale γ′ phases, thereby significantly enhancing creep resistance. As illustrated in Figure 5d, creep cavities nucleate in the eutectic region adjacent to the primary dendrite stem and subsequently extend through the interdendritic pathways. As depicted in Figure 5e, the L specimen exhibits an elliptical fracture surface, arising from the interplay between lattice rotation and crystal anisotropy during deformation. This trait is comparable to the characteristics of single-crystal superalloys [32,33,34]. As shown in Figure 5f, numerous creep cavities can be observed on the fracture surface of the L specimen, with secondary cracks around the cavities. Under high magnification, many oxidized granular particles and local dimples can be seen. From Figure 5g,h, it can be observed that the dendritic stem of the L specimen is parallel to the principal stress direction, and significant plastic deformation has occurred in the dendritic stem near the fracture. Cracks also preferentially propagate along the interdendritic path, and after forming larger cracks, they can penetrate through the dendritic stem. The fracture surface is overall at a 45° angle to the principal stress direction.

**Figure 6:**
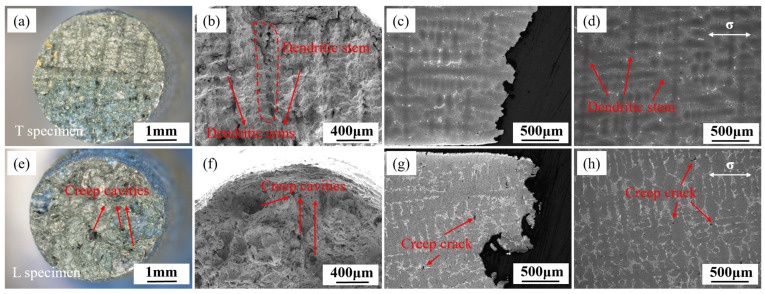
Fracture surface morphology (**a**,**b**,**e**,**f**) and microstructure (**c**,**d**,**g**,**h**) near the fracture of T specimens (**a**–**d**) and L specimens (**e**–**h**) after rupture at 1070 °C/50 MPa.

The rupture fracture surface morphology and microstructure near the fracture of T and L specimens under the condition of 1070 °C/50 MPa are shown in Figure 6. For the T specimens, a dendritic morphology can be observed, which is formed by the fracture propagating along the interdendritic path near the dendrite stem and dendrite arms, indicating that the T specimen exhibits more pronounced interdendritic fracture characteristics at 1070 °C. The fracture surface of the L specimen exhibits multiple creep cavities, as illustrated in Figure 6e,f. Observing the microstructure near the fracture from the L section, it can be seen that creep cracks are distributed in the eutectic regions, as shown in Figure 6g,h, indicating that the L specimen also nucleates interdendritic cracks. After forming in the secondary dendrite regions, the creep cracks propagate perpendicular to the stress direction and eventually cut through the dendrite stem. In comparison, the fracture surface of the T specimen is flatter, while the L specimen shows more interdendritic characteristics and significant necking.

## 4. Discussion

### 4.1. Deformation Mechanisms

#### 4.1.1. Deformation Mechanisms of Creep at 980 °C/140 MPa

**Figure 7:**
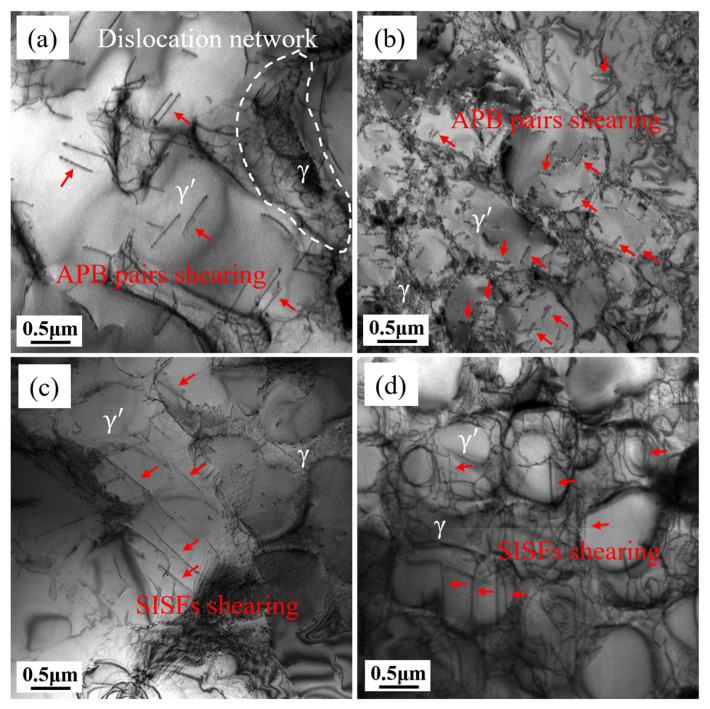
Dislocation structures of L (**a**,**b**) and T (**c**,**d**) specimens: 1% deformation (**a**,**c**); after fracture (**b**,**d**).

L and T specimens were subjected to creep tests at 980 °C/140 MPa, and samples were taken for observation under TEM after 1% deformation and after fracture, as shown in Figure 7. Dislocation networks become visible within the γ channels of both L and T specimens when subjected to 1% deformation, with lower dislocation density in the γ′ precipitate. Dislocations of different orientations in the γ matrix interweave to form dislocation networks, and when dislocations reach the γ/γ′ interface, they are impeded by γ′ and accumulate there. A greater driving force is required for some dislocations to cut through the L1_2_-ordered γ′ precipitate along a certain orientation. Observing the dislocations in the γ′ precipitate of L and T specimens, the dislocations in the γ′ precipitate of the L specimen are shorter, appearing in pairs with wider APBs region, while those in the T specimen are thinner and longer. The width of the APB or SISF region is determined by the APB energy and SISF energy. Since the APB energy is relatively high, typically 100–300 mJ/m^2^, APBs tend to be wider and appear as uniform stripe contrast in TEM bright-field images. The SISF fault energy is lower, usually 20–80 mJ/m^2^, resulting in narrower fault regions. In TEM, SISF appears as thin, elongated stripes, and its width is sensitive to diffraction conditions [35,36]. Therefore, based on the morphology of dislocation lines, it can be preliminarily inferred that APBs shearing is dominant in the L specimen, while SISFs shearing is dominant in the T specimen. Further dislocation characterization is needed to elucidate the different dislocation motion mechanisms in L and T specimens. Figure 7b,d show the dislocation morphology after fracture, which is similar to that at 1% deformation, with short paired dislocation lines in the γ′ precipitate of the L specimen and long dislocation lines in the T specimen. In the post-fracture microstructure of the L specimen, the γ′ precipitate is severely sheared, and the dislocations in the γ channels are complex and intertwined, making it difficult to distinguish individual dislocation lines. Compared with the L specimen, the post-fracture microstructure of the T specimen has more uniform dislocation line directions, implying that slip system activation is less pronounced in the T specimen.

**Figure 8:**
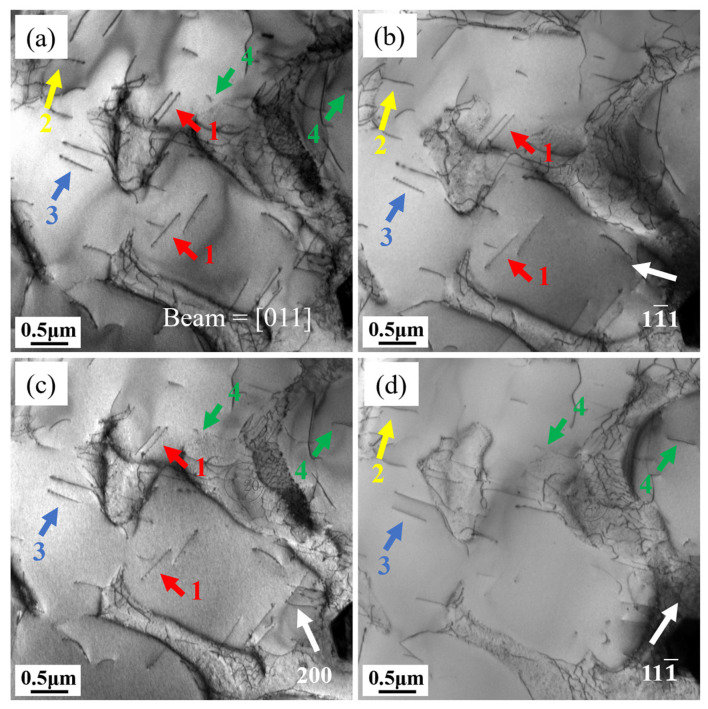
Characterization of the Burgers vectors associated with dislocations 1–4 in the L specimen under distinct diffraction scenarios: (**a**) Beam = [011], (**b**) g: 1$\bar{1}1$, (**c**) g: 200, (**d**) g: 11$\bar{1}$.

To examine the characteristics of dislocations in L and T specimens, multiple two-beam diffraction conditions were employed. These conditions enabled the visualization of dislocations in creep samples subjected to 1% deformation, analyzed under varying zone axes and g vectors. Figure 8 displays the dislocation arrangement in the L sample along the [011] zone axis. Distinct Burgers vectors of dislocations result in observable contrast variations (visible/invisible) under different g vectors, with Table 2 detailing the extinction behavior of dislocations 1–4 under specific diffraction conditions and their corresponding Burgers vectors. The Burgers vector of dislocation 3 could not be derived due to insufficient conditions, but based on the morphology of the dislocation line and its paired appearance, it can be inferred that dislocation 3 also belongs to the <110>{111} slip system. In precipitation-strengthened nickel-based superalloys, γ′ precipitates can effectively impede dislocation motion. The way dislocations cut through γ′ precipitates depend on temperature, stress, the size of the γ′ precipitates, APB energy, stacking fault energy, or their combinations [37]. γ′ precipitates have an L1_2_ ordered structure. When dislocations cut into the γ′ precipitates, they disrupt this ordered structure and generate an additional APB or SISF. When a subsequent dislocation cuts into the γ′ precipitates, the APB or SISF closes, and the γ′ precipitates maintain their L1_2_ ordered structure. Therefore, dislocations cutting through the γ′ precipitates always appear in pairs [38]. Figure 8 reveals that the L specimen exhibits dislocation arrangements occurring in pairs. Based on the extinction patterns in the two-beam condition, dislocations 1, 2, and 4 are identified as a/2<110> partial dislocations. The a/2<110> partial dislocations form a pair as a result of the dissociation of a complete a<110> dislocation. Hence, it follows that the dislocation shearing of γ′ precipitates in the L specimen is mainly due to APB shearing, with the dislocation reaction being:

a<110> → a/2<110> + APB + a/2 <110>

The stress direction of the L specimen is [001]. The Schmid factors for the 12 slip systems in <110>{111} can be calculated by m=cos⁡φcos⁡λ, where φ represents the angle formed by the slip plane’s normal direction and the axis of the applied stress, while λ denotes the angle between the slip direction and the stress axis. When the value of m is larger, the resolved shear stress is greater, and the slip system is more easily activated. Therefore, the priority of slip system activation can be predicted based on the value of m. Calculations of the Schmid factors for the L specimen reveal that eight <110>{111} slip systems have the maximum m value of 0.408 simultaneously. Thus, in the L specimen, eight <110>{111} slip systems are predominantly activated. The simultaneous activation of slip systems in different directions is conducive to uniform plastic deformation of the alloy, resulting in a higher elongation at fracture for the L specimen. Additionally, since the activation critical value for the <110>{111} slip systems is relatively low, the γ′ precipitates exhibit lower resistance to dislocation shearing, leading to lower creep deformation resistance and a higher minimum creep rate.

**Figure 9:**
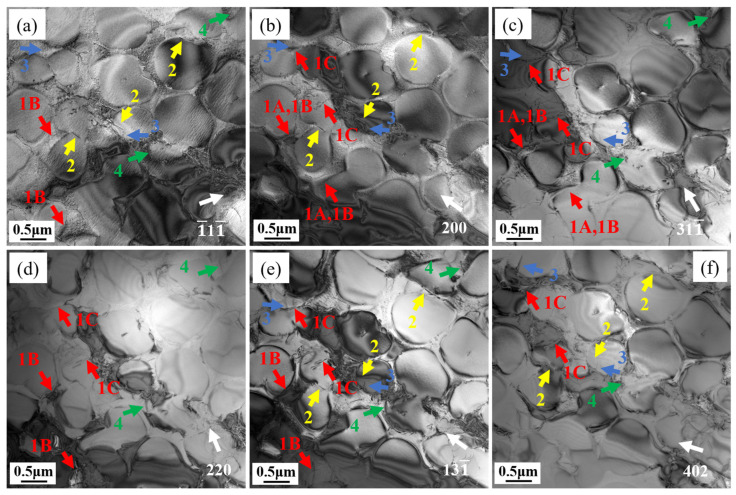
Characterization of the Burgers vectors associated with dislocations 1–4 in the T specimen under distinct diffraction scenarios: (**a**) g: $\bar{1}$ 1$\bar{1}$, (**b**) g: 200, (**c**) g: 31$\bar{1}$, Beam = [011]; (**d**) g: 220, (**e**) g: 13$\bar{1}$, (**f**) g: 402, Beam = [$\bar{1}$12].

The dislocation morphology of the T specimen under [011] and [112] zone axes are show in Figure 9. Table 3 summarizes the visibility of dislocations 1–4 under different diffraction conditions. Interestingly, dislocation 1 can be divided into three types: 1A, 1B, and 1C. These three types of dislocations show different extinction patterns under different g vectors, indicating that they have different Burgers vectors. When observed along the [011] zone axis with g = 111, dislocations 1A and 1C exhibit invisibility, whereas dislocation 1B remains visible. Switching to the [112] zone axis under g = 200 or g = 131, dislocation 1A becomes invisible, while 1B and 1C are clearly detectable. However, with g = 402 under the same axis, dislocations 1A and 1B are no longer visible, leaving only 1C observable. By applying the extinction criteria, the Burgers vectors of dislocations 1–4 were determined and are summarized in Table 3.

As shown in Table 3, in addition to <112>{111} dislocations, there are also some <110>{111} dislocations in the T specimen. Previous studies have found that at 850 °C, the creep deformation of the T specimen of this alloy is mainly due to <112>{111} dislocations shearing through γ′, forming Shockley partial dislocations and SISFs [24]. These SISFs cross-slip on close-packed planes and widen to form slip bands. The creep deformation mechanism of the T specimen at 980 °C is similar to that at 850 °C, but no slip band were observed at 980 °C, and the quantity of <110>{111} dislocations increased. Therefore, it can be reasonably inferred that due to the increase in temperature, the atomic diffusion rate increases, and the dislocation slip resistance decreases. The coexistence of dislocations in the <112>{111} and <110>{111} slip systems seems to indicate that both APB shearing and SISF shearing exist in the creep process of the T specimen, or that there is some kind of transformation relationship between the two. The research by Yang H. et al. [39] shows that SISFs can be dissociated from APBs, and the dissociation reaction can be described using the equation shown below:

a<110> → a/2<110> +APB + a/2<110>

a/2<110> → a/3<112> + SISF + a/6<112>

Combining the observed Burgers vectors of dislocations 1A, 1B, and 1C, and based on the vector relationships and energy criteria of dislocation reactions, the following reactions can be inferred between these three types of dislocation lines:

$$a[10\bar{1}]\to a/2[10\bar{1}]+APB+a/2[10\bar{1}]$$

$$a/2[10\bar{1}]\to a/3[1\bar{1}\bar{2}]+SISF+a/6[121]\;for\;the\;leading\;partial$$

$$a/2[1\bar{1}0]\to a/6[1\bar{2}1]+SISF+a/3[11\bar{2}]\;for\;the\;trailing\;partial$$

Based on the above analysis, it can be reasonably inferred that during the creep process of the T specimen at 980 °C, APB pairs are initially formed. Due to crystal orientation, some APB pairs find it difficult to move in the <110>{111} slip system. As dislocations pile up and accumulate sufficient energy, the APB pairs dissociate. The dissociation process begins with an a/2 [10$\bar{1}$] leading partial dislocation dissociating to form a super-Shockley dislocation a/3 [1$\bar{1}\bar{2}$] and a Shockley dislocation a/6 [121]. Subsequently, the trailing a/2 [1$\bar{1}$0] partial dislocation dissociates again to form a super-Shockley dislocation a/3 [11$\bar{2}$] and a Shockley dislocation a/6 [1$\bar{2}$1]. The two adjacent Shockley partial dislocations eventually annihilate at the γ/γ′ interface, leaving two super-Shockley dislocations: a/3 [1$\bar{1}\bar{2}$] and a/3 [11$\bar{2}$].

The way dislocations cut through γ′ precipitates depend on the relative magnitudes of the APB energy and the stacking fault energy. Many studies [40,41,42,43,44] have shown that as temperature increases, APB energy decreases, while stacking fault energy increases. SISF shearing is more common at low temperatures, while APB shearing dominates at high temperatures. Therefore, for the T specimen, as the temperature rises, the way dislocations cut through γ′ precipitates gradually shift from SISF shearing to APB shearing. At the intermediate temperature of 980 °C, a combination of SISF and APB shearing occurs. Since the <112> direction in the <112>{111} slip system is not a close-packed direction of the L1_2_ ordered structure, atomic migration is difficult, resulting in higher creep deformation resistance and lower minimum creep rates. Additionally, the limited activation of the <112>{111} slip systems results in inhomogeneous plastic deformation, which reduces the elongation at fracture. In contrast, the L specimen exhibits a higher propensity for the <110>{111} slip system to be activated and multiple orientations of APB pairs shear simultaneously, which is conducive to uniform plastic deformation. This results in lower creep deformation resistance, higher minimum creep rates, along with enhanced elongation.

#### 4.1.2. Deformation Mechanisms of Rupture at 1070 °C/50 MPa

**Figure 10:**
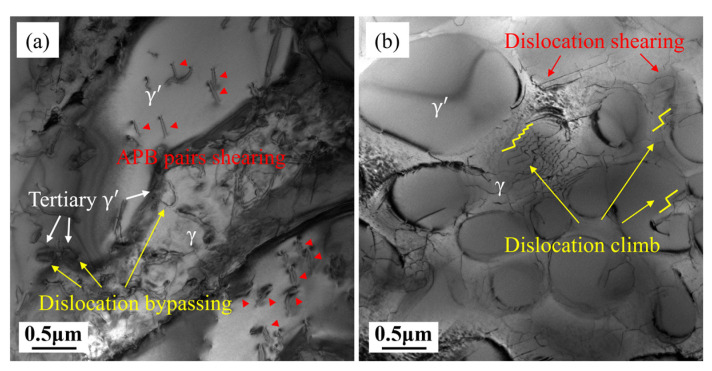
Dislocation structures at 1/4 rupture life in L (**a**) and T (**b**) specimens.

L and T specimens were subjected to rupture tests at 1070 °C/50 MPa, and samples were taken for observation under TEM at 1/4 of the rupture life, as shown in Figure 10. The γ′ precipitates in the L specimen have undergone significant coarsening and rafting at 1/4 life, as shown in Figure 10a. The dislocation density in the γ channels is high, and the dislocation network structure is no longer visible. Tertiary γ′ precipitates have also formed within the γ channels, with dislocation lines bypassing the tertiary γ′ to form ring-shaped dislocation. Clear APB dislocation pairs are visible in the γ′ precipitates, and the direction of the dislocation lines indicates that multiple slip systems are activated at 1070 °C. Therefore, deformation in the L specimen at 1070 °C is mainly composed of dislocation shearing of secondary γ′ and dislocation bypassing of tertiary γ′. The γ′ in the T specimen has also undergone a certain degree of coarsening, as illustrated in Figure 10b, but the rafting is significantly less than that in the L specimen. The dislocation morphology is also distinctly different from that in the L specimen. In the γ channels, zigzag dislocation lines composed of mutually perpendicular line segments can be observed, which, based on the literature experience [45,46,47], are typical climb dislocation lines. Long dislocation lines cutting through the γ′ precipitates can also be seen. Therefore, deformation in the T specimen at 1070 °C is mainly from dislocation climb in γ channels and dislocation shearing through γ′ precipitates.

#### 4.1.3. Summary of Creep–Rupture Deformation Mechanisms

**Figure 11:**
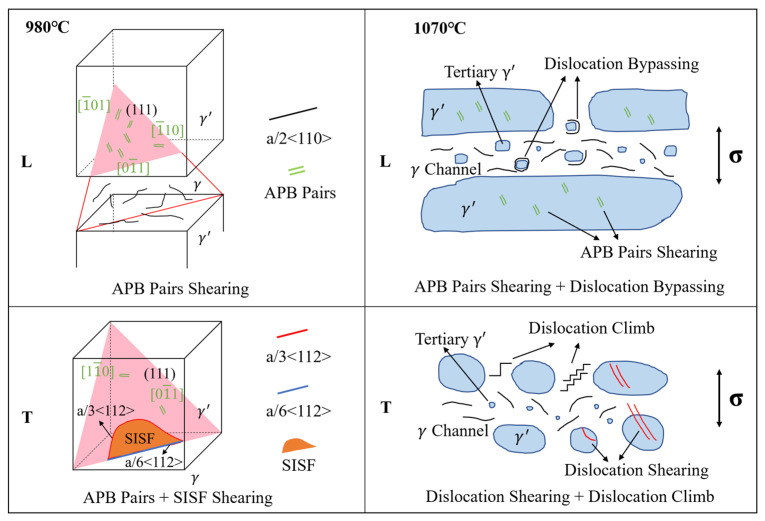
Diagrammatic representation of the creep mechanisms occurring in L and T specimens at 980 °C and 1070 °C.

Drawing from the preceding analysis, the mechanisms governing deformation during the creep/rupture process of the DS superalloy at 980 °C and 1070 °C are summarized, as illustrated in Figure 11. At 980 °C, the creep mechanism of the L specimen is dominated by the stimulation of several <110>{111} slip systems, with APB pairs shearing through the γ′ precipitate. The T specimen features a mixed activation of <110>{111} and <112>{111} slip systems, with both APB and SISF shearing occurring simultaneously. At 1070 °C, the creep mechanism of the L specimen consists of dislocation shearing of γ′ and dislocation bypassing of tertiary γ′. The T specimen involves dislocation climb in the γ channels bypassing the γ′ and dislocation shearing through the γ′.

### 4.2. Microstructural Evolution

**Figure 12:**
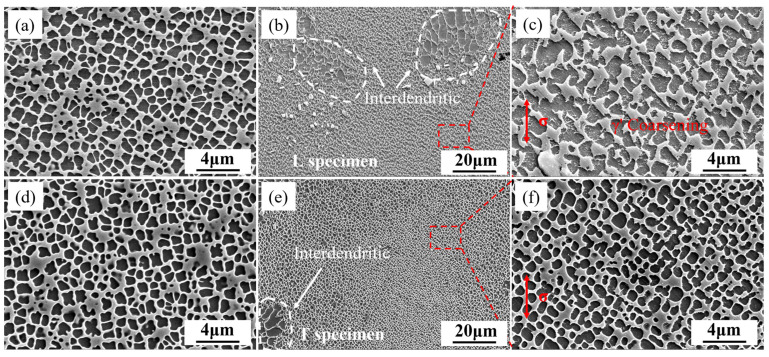
Microstructural evolution of L (**a**–**c**) and T (**d**–**f**) specimens at 980 °C: original state (**a**,**d**) and after fracture (**b**,**c**,**e**,**f**).

At elevated temperature, the degradation of the γ′ in DS superalloys occurs due to the synergistic effect of multiple mechanisms, including spheroidization, coarsening, rafting, elemental diffusion, and interface failure. Understanding the microstructural evolution patterns of the alloy and implementing a coordinated design of composition, process, and microstructure can effectively delay the degradation of the γ′ phase and enhance the high-temperature performance of the alloy. The microstructures of the L and T specimens after creep at 980 °C/140 MPa are illustrated in Figure 12. After more than 200 h of creep, the microstructures of the L and T specimens changed little, which can be seem in Figure 12c,f. The γ′ in the L specimen coarsened to some extent, from the original size of 810 ± 130 nm to 1620 ± 270 nm, while the γ′ in the T specimen showed no significant coarsening. Neither the T nor the L specimens showed significant rafting. According to the lattice mismatch of γ/γ′ calculated in previous work [24], the alloy exhibits a near-zero lattice mismatch at 980 °C, so the interfacial mismatch stress caused by the lattice constant is very small, and no significant rafting occurs in the alloy under different orientations. Compared with the original microstructure in Figure 2, it can be found that the skeletal carbides in the interdendritic regions of both the L and T specimens have disappeared, which were common in the original microstructure, indicating that the carbides in the alloy have dissolved at 980° C. The skeletal carbides form a continuous network in the interdendritic regions and can inhibit dislocation slip through pinning, thereby improving the alloy’s ability to resist creep under elevated temperatures. With the decomposition of the skeletal carbides, the alloy microstructure degrades, the creep resistance decreases, and the creep rate increases.

**Figure 13:**
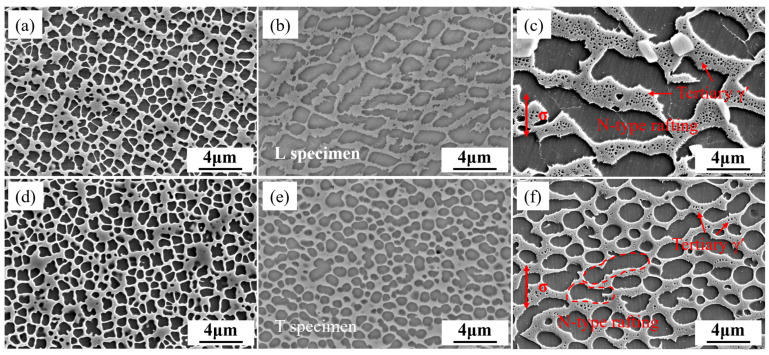
Microstructural evolution of L (**a**–**c**) and T (**d**–**f**) specimens at 1070 °C: original state (**a**,**d**), 1/4 life (**b**,**e**) and after fracture (**c**,**f**).

The microstructures of the L and T specimens at 1/4 life and after rupture at 1070 °C/50 MPa are shown in Figure 13. The L specimen has already undergone significant rafting at 1/4 life, with the γ channels also coarsening and some tertiary γ′ precipitates forming in the coarsened γ channels. This rafting and the precipitation of tertiary γ′ phases are further enhanced in the post-fracture microstructure. Stress-induced oriented diffusion causes the γ′ phase to redistribute in the matrix channels, with dissolution in high-stress regions and precipitation in low-stress regions. At 1070 °C, the alloy has a negative γ/γ′ lattice mismatch, and under tensile stress, the γ′ reorients itself orthogonally to the stress axis, resulting in N-type rafting. In our previous studies, we have found that the L specimen of this alloy forms P-type rafting at 850 °C, which arises from the positive γ/γ′ lattice misfit.

The rafting in the T specimen is not significant, as shown in Figure 13e,f. The γ′ phase in the T specimen shows a certain degree of spheroidization at 1/4 life, and further coarsening occurs in the post-fracture microstructure. Accompanying the coarsening of the γ channels, tertiary γ′ also form in the T specimen, with some coarsened γ′ phases merging to form slight N-type rafting. The γ′ phase gradually undergoes spheroidization and coarsening with increasing time, which is a clear characteristic of Ostwald ripening. Therefore, the ripening rate constant for the T specimen can be derived based on the Ostwald ripening theory [48,49]. Based on Fick’s law and the Gibbs–Thomson effect, the growth rate of a single particle can be expressed as:

(2) $$\frac{dr}{dt}=\frac{2D\gamma \Omega^{2}C_{\infty }}{kT}\cdot \frac{1}{r^{2}}(\frac{1}{r_{c}}-\frac{1}{r})$$

where $r_{c}$ is the critical radius, defined as the radius of particles in the system that neither grow nor dissolve; D is the solute diffusion rate, in m^2^/s; γ is the interfacial energy, in mJ/m^2^; Ω presents atomic volume, in m^3^; $C_{\infty }$ stands for the equilibrium concentration, defined as the solute equilibrium concentration at a flat interface (infinite particle size); k is the Boltzmann constant; and T is the thermodynamic temperature, in K.

Provided that the overall solute concentration within the system remains constant and the combined volume of particles is unchanged, we have:

(3) $$\int_{0}^{\infty }r^{3}f(r,t)dr=C$$

C is a constant. Combining the growth rate equation, the evolution law of the critical radius can be derived as:

(4) $$r_{c}^{3}(t)=r_{c}^{3}(0)+Kt$$

The size of γ′ in the T specimen at 1/4 rupture life and after fracture was measured. By fitting the slope of r^3^ ∝ t, the Ostwald ripening rate constant for the T specimen was obtained as 1.04 × 10^−20^ m^3^/h.

**Figure 14:**
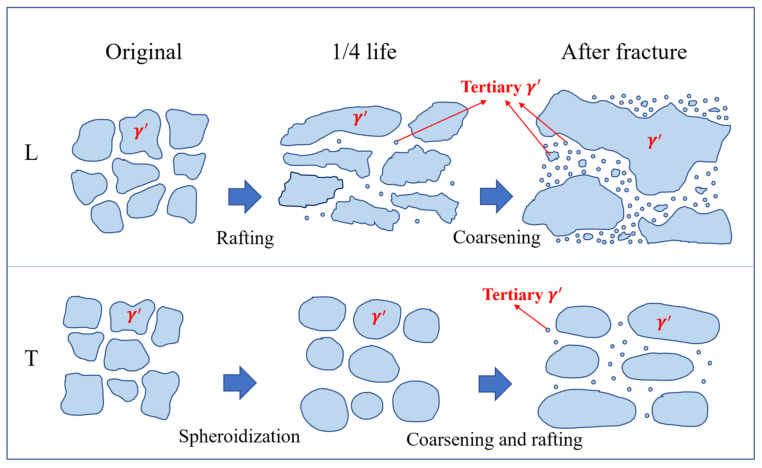
Schematic diagrams of microstructural evolution at 1070 °C.

Based on the above discussions, the microstructural evolution patterns of the DS superalloy during the rupture process at 1070 °C are summarized, and the schematic diagram of microstructural evolution during the rupture process at 1070 °C is shown in Figure 14. Rafting in the L specimen had already formed at 1/4 life, with the rafting direction orthogonal to the stress axis, forming N-type rafting. Nucleation of tertiary γ′ was initiated within γ channels. Subsequently, as the rupture time increased, both the rafted γ′ and the γ channels continued to coarsen, with a large number of tertiary γ′ forming in the coarsened γ channels and a tendency to grow larger. The T specimen exhibited significant Ostwald ripening characteristics, with the γ′ phase gradually undergoing spheroidization and coarsening over time. Its Ostwald ripening rate constant was 1.04 × 10^−20^ m^3^/h, and a certain degree of N-type rafting occurred by the time of fracture, with relatively fewer tertiary γ′ precipitates in the γ channels.

## 5. Conclusions

This study investigates the role of crystallographic orientation in the high-temperature creep properties of DS superalloys. Tests for creep and rupture behavior were carried out at 980 °C and 1070 °C. The microstructures of samples during deformation and after fracture were characterized to investigate the mechanisms underlying creep deformation and the patterns of microstructural evolution, leading to the following conclusions:

The T specimen exhibits a lower minimum creep rate at 980 °C, indicating higher creep resistance, which can provide a basis for the design of some hot section components that require high creep resistance and small deformation.

(1) At 980 °C, the primary creep mechanism in the L specimen involves the operation of multiple <110>{111} slip systems, accompanied by APB pairs cutting through the γ′ phase. In contrast, the T specimen exhibits a combination of <110>{111} and <112>{111} slip system activation, where both APB and SISFs shearing take place concurrently. When the temperature increases to 1070 °C, the L specimen’s deformation behavior shifts to a combination of γ′ dislocation shearing and tertiary γ′ bypassing by dislocations. Meanwhile, the T specimen demonstrates dislocation climb within the γ channels along with dislocation shearing of the γ′.
(2) Under creep conditions at 980 °C, neither the L nor T specimens demonstrated notable rafting behavior. However, the γ′ phase in the L specimen underwent coarsening, increasing from an initial size of 810 nm to 1470 nm. When subjected to rupture testing at 1070 °C, the L specimen displayed pronounced N-type rafting, accompanied by the formation of numerous tertiary γ′ within the widened γ channels. In contrast, the T specimen revealed distinct Ostwald ripening features, with a calculated ripening rate constant of 1.04 × 10^−20^ m^3^/h.
(3) Nevertheless, this research is not without its limitations. One such constraint is the anisotropy of creep was not correlated with life prediction. In fact, a unified life prediction model for different orientations can be established through viscoplasticity theory. These also point the direction for our future research.

## Figures and Tables

**Table 1:** The creep life and minimum creep rates across various stress levels at 980 °C.

Specimens		110 MPa	140 MPa	160 MPa	180 MPa
T	Creep life, h	794 ± 23	246 ± 19	86 ± 3.6	56 ± 1.8
	minimum creep rates, %/h	0.0061	0.0113	0.0184	0.0599
L	Creep life, h	1456 ± 37	272 ± 1.2	194 ± 7.5	64 ± 0.6
	minimum creep rates, %/h	0.0086	0.0339	0.0438	0.1285

**Table 2:** Dislocation visibility in the L specimen under differing diffraction conditions.

Beam	g	1	2	3	4
[011]	1$\bar{1}$1	√	√	√	×
	200	√	×	√	√
	11$\bar{1}$	×	√	√	√
Burgers vectors b		$\pm \frac{a}{2}[101]$ *or* $\pm \frac{a}{2}[\bar{1}10]$	$\pm \frac{a}{2}[0\bar{1}1]$	Unknow	$\pm \frac{a}{2}[10\bar{1}]$

Note: × represents invisible, √ represents visible.

**Table 3:** Dislocation visibility in the T specimen under differing diffraction conditions.

Beam	g	1A	1B	1C	2	3	4
[011]	$11\bar{1}$	×	√	×	√	√	√
	200	√	√	√	√	√	×
	$31\bar{1}$	√	√	√	×	√	√
[$\bar{1}$12]	220	×	√	√	×	×	√
	$13\bar{1}$	×	√	√	√	√	√
	402	×	×	√	√	√	√
Burgers vectors b		$\pm \frac{a}{3}[1\bar{12}]$	$\pm \frac{a}{3}[11\bar{2}]$	$\pm \frac{a}{2}[10\bar{1}]$	$\pm \frac{a}{3}[1\bar{1}2]$	$\pm \frac{a}{2}[1\bar{1}0]$	$\pm \frac{a}{2}[01\bar{1}]$

Note: × represents invisible, √ represents visible.

## Data Availability

The original contributions presented in the study are included in the article. Further inquiries can be directed to the corresponding author.
